# Determinants of pegivirus persistence, cross-species infection, and adaptation in the laboratory mouse

**DOI:** 10.1371/journal.ppat.1012436

**Published:** 2024-08-28

**Authors:** Kylie Nennig, Satyapramod Murthy, Sara Maloney, Teressa M. Shaw, Mark Sharobim, Eduard Matkovic, Simi Fadiran, Malorie Larsen, Mitchell D. Ramuta, Arthur S. Kim, John R. Teijaro, Joe Grove, Matthew Stremlau, Himanshu Sharma, Sheetal Trivedi, Michael J. Blum, David H. O’Connor, Jennifer L. Hyde, Jack T. Stapleton, Amit Kapoor, Adam L. Bailey

**Affiliations:** 1 Department of Pathology and Laboratory Medicine, University of Wisconsin–Madison School of Medicine and Public Health, Madison, Wisconsin, United States of America; 2 Center for Vaccines and Immunity, The Research Institute at Nationwide Children’s Hospital, Columbus, Ohio, United States of America; 3 Department of Immunology and Microbiology, The Scripps Research Institute, San Diego, California, United States of America; 4 Department of Chemistry, The Scripps Research Institute, San Diego, California, United States of America; 5 MRC-University of Glasgow Center for Virus Research, Glasgow, United Kingdom; 6 Brain Science Institute, Johns Hopkins University School of Medicine, Baltimore, Maryland, United States of America; 7 Department of Ecology & Evolutionary Biology, University of Tennessee, Knoxville, Tennessee, United States of America; 8 Department of Microbiology, University of Washington, Seattle, Washington, United States of America; 9 Department of Internal Medicine, Microbiology & Immunology, University of Iowa and Iowa City Veterans Affairs Healthcare System, Iowa City, Iowa, United States of America; 10 Department of Pediatrics, College of Medicine and Public Health, Ohio State University, Columbus, Ohio, United States of America; The University of Chicago, UNITED STATES OF AMERICA

## Abstract

Viruses capable of causing persistent infection have developed sophisticated mechanisms for evading host immunity, and understanding these processes can reveal novel features of the host immune system. One such virus, human pegivirus (HPgV), infects ~15% of the global human population, but little is known about its biology beyond the fact that it does not cause overt disease. We passaged a pegivirus isolate of feral brown rats (RPgV) in immunodeficient laboratory mice to develop a mouse-adapted virus (maPgV) that established persistent high-titer infection in a majority of wild-type laboratory mice. maRPgV viremia was detected in the blood of mice for >300 days without apparent disease, closely recapitulating the hallmarks of HPgV infection in humans. We found a pro-viral role for type-I interferon in chronic infection; a lack of PD-1-mediated tolerance to PgV infection; and multiple mechanisms by which PgV immunity can be achieved by an immunocompetent host. These data indicate that the PgV immune evasion strategy has aspects that are both common and unique among persistent viral infections. The creation of maPgV represents the first PgV infection model in wild-type mice, thus opening the entire toolkit of the mouse host to enable further investigation of this persistent RNA virus infections.

## Author summary

Chronic viral infections take an enormous toll on human health: over 300 million people live with chronic hepatitis B, hepatitis C, or HIV. However, animal models of chronic viral infection are largely confined to nonhuman primates. As such, the vast resources of the laboratory mouse (i.e., transgenic animals, reagents, increased statistical power) have been underutilized to study chronic viral infection. Here, we adapted a rat pegivirus (RPgV) to infect the laboratory mouse. Once adapted to this new host, the mouse-adapted pegivirus (maPgV) causes persistent high-titer viremia in wild-type (i.e., not immunocompromised) laboratory mice for hundreds of days. This new model of persistent viral infection exhibited unique properties, including a lack of disease in immunocompromised mice or mice with a hyperactive immune system. The creation of maPgV represents the first pegivirus infection model in wild-type mice, thus opening the entire toolkit of the mouse host to enable further investigation of persistent RNA infections.

## Introduction

The *Pegivirus* genus is one of four genera in the *Flaviviridae* family of enveloped +ssRNA viruses, which also includes the *Orthoflavivirus* genus (*e*.*g*., Dengue, Zika, Yellow fever, and West Nile viruses), the *Hepacivirus* genus (*e*.*g*., hepatitis C virus), and the *Pestivirus* genus (*e*.*g*., bovine diarrhea virus) [1]. Human pegivirus (HPgV or HPgV-1, formerly known as GB virus C and also as Hepatitis G virus) causes long-lasting infection in ~15% of the global human population, making it the most prevalent blood-borne RNA virus infection (~10-fold more common than hepatitis C virus, HCV) [2]. HPgV is transmitted via sexual, blood-borne, and vertical routes, and causes a persistent viremia (as defined by the presence of ~1×10^6−8^ viral genomes per mL of serum) that can last for decades, although ‘spontaneous’ clearance is well documented [3]. Unlike other members of the *Flaviviridae*, HPgV does not cause overt disease. However, two noteworthy associations with HPgV infection have been identified: in large studies of HIV+ patients, HPgV co-infection is associated with a significant reduction in all-cause mortality and HIV-induced pathological immune activation, implying that HPgV infection is a positive prognostic indicator in the context of HIV infection [4–7]. Additionally, a weak but statistically-significant correlation exists between chronic HPgV infection and the development of non-Hodgkin B cell lymphoma [8].

Viruses capable of persistently infecting an immunocompetent host have sophisticated strategies for evading the host immune response including infection of immune-privileged sites (*e*.*g*., the central nervous system [CNS]), latency, and the accumulation of mutations in key immune-targeting epitopes (*i*.*e*., immune escape) [9,10]. However, PgVs appear to employ none of these strategies, causing high-titer viremia for years without accumulating mutations indicative of immune escape [11,12]. Investigations into this apparently novel mechanism of immune avoidance have been hindered by the lack of robust culture systems or small animal models for studying PgV in the laboratory.

In this study, we created a lab mouse model of PgV infection by adapting a RPgV isolated from a feral-brown rat (*Rattus norvegicus*) captured in New Orleans. Adaptation to the mouse host initially required defective innate immunity (*STAT1*^-/-^ or *IFNAR*^-/-^), which resulted in the outgrowth of a minimally-mouse-adapted PgV containing just a single nonsynonymous mutation in the E2 glycoprotein. Upon passage into wild-type (WT) mice, this virus rapidly and consistently accumulated an additional 6 nonsynonymous mutations (concentrated in structural proteins) and 6 synonymous mutations (concentrated in non-structural proteins), resulting in a “fully” mouse-adapted PgV (maPgV). The kinetics of maPgV infection in WT mice was highly reproducible, with an initial peak of viremia at ~15 days post infection (dpi) followed by a steady decrease in viral load until ~100 dpi, at which a viremic set-point was established that lasted >300 days for ~90% of infected animals. Infection of knockout mice lacking various immune genes showed that *RAG*^-/-^ mice maintained significantly higher levels of chronic-phase viremia, while *IFNAR*^-/-^ mice exhibited higher acute-phase viral loads but eventually cleared viremia at a significantly higher rate than WT mice. *PD1*^-/-^ mice displayed a blunted peak of maPgV viremia but established chronic viremic set-points similar to those of WT mice. Investigation of correlates of immunity in rare WT mice that controlled maPgV infection revealed distinct “pathways” to maPgV immunity, including cellular immunity that can be passively transferred between animals. Altogether, our creation of a mouse-adapted pegivirus greatly expands the questions that can be posed to interrogate the unique biology of this enigmatic group of viruses.

## Results

### Disabling the type-I-interferon–Stat1 pathway enables adaptation of RPgV to mice

**Fig 1:**
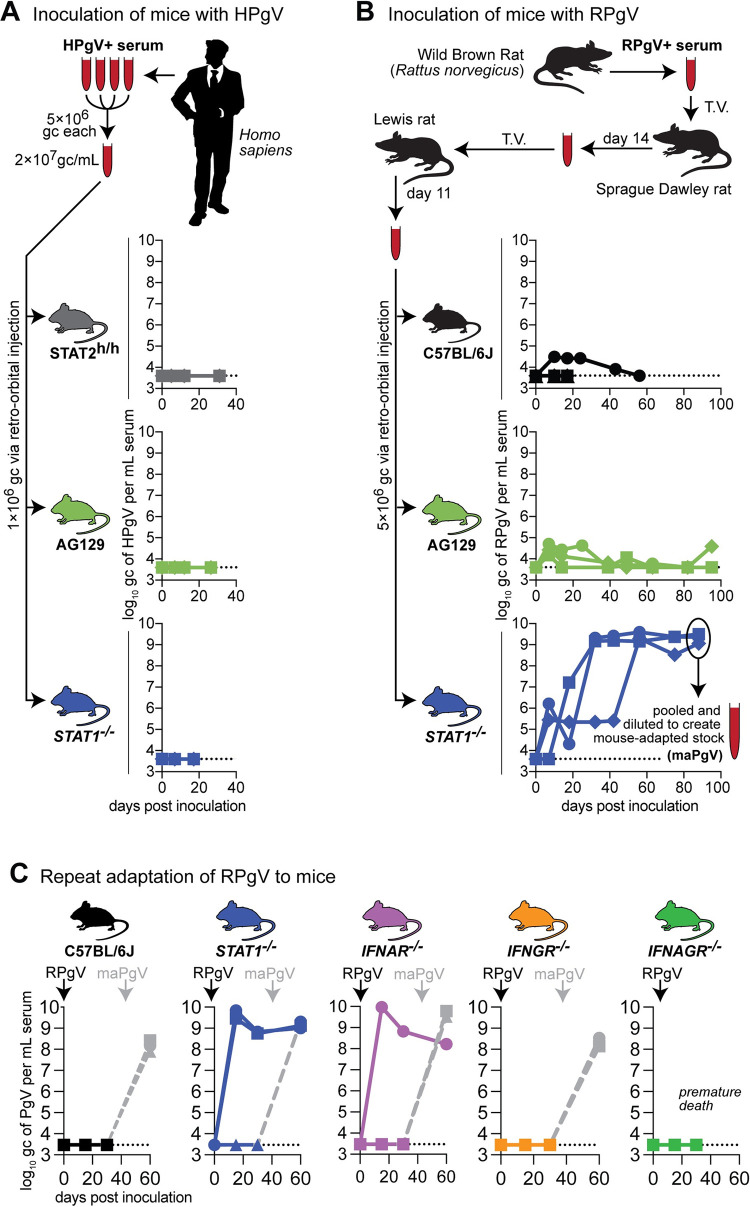
Disabling the interferon-alpha–Stat-1 pathway enables adaptation of RPgV to mice. **(A)** Pooled serum from multiple donors was inoculated into mice via retro-orbital injection: human STAT2-knock-in c57BL6/J mice (grey), *IFNAGR*^-/-^ “AG”129 mice (green), or *STAT1*^-/-^ mice (blue), n = 3 per group, with unique symbols for each individual. Serum HPgV loads were measured using an HPgV-specific RT-qPCR assay, with the dashed line demarcating the limit of detection. **(B)** Serum containing RPgV was inoculated into mice via retro-orbital injection: wild-type c57BL6/J (black), *IFNAGR*^-/-^ “AG”129 mice (green), and *STAT1*^-/-^ mice (blue), n = 3 per group, with unique symbols for each individual. Serum from the 3 STAT1-knockout mice was collected and pooled to create a large “mouse-adapted” pegivirus (maPgV) virus stock. **(C)** Repeat of the RPgV adaptation study was performed via via retro-orbital injection of RPgV into: human wild-type c57BL6/J mice (black), *STAT1*^-/-^ mice (blue), *IFNAR*^-/-^ “AG”129 mice (purple), *IFNGR*^-/-^ “AG”129 mice (yellow), or *IFNAGR*^-/-^ “AG”129 mice (green), n = 3 per group. Re-inoculation with maPgV to examine RPgV-induced immunity is shown in gray.

Despite the prevalence of PgVs in mammals, naturally-occurring PgV infection of a house mouse (*Mus musculus*) has not, to our knowledge, been described. We therefore began by inoculating various immunocompromised mice––Interferon (IFN)α and γ receptor-deficient (a.k.a “AG129”), *STAT1*^-/-^ c57BL6/J mice, and c57BL6/J mice expressing human STAT2 (*STAT2*^h/h^)––with pooled serum from HPgV-1-infected humans. None of these animals supported HPgV-1 infection, as determined by HPgV-1-specific RT-qPCR on serum (**Fig 1A**). PgVs appear to have a narrow host-species tropism: HPgV-1 will infect chimpanzees but not macaques [2,11]; however, baboon PgVs readily infect macaques [12]. Given this, we inoculated wild-type (WT) and immunocompromised mouse strains with serum containing a PgV isolated from another rodent: the brown rat (*Rattus norvegicus*). Low levels of viremia were detected in WT c57BL6/J and AG129 mice, but RPgV never achieved sustained titers of greater than 1×10^5^ genome copies (gc) per mL of serum in these animals (**Fig 1B**). In contrast, RPgV titers fluctuated in three *STAT1*^*-/-*^ mice until ~60 days post-inoculation (dpi), after which RPgV titers rose to ~1×10^9^ gc/mL of serum. This high titer was maintained until 88 dpi, at which point mice were euthanized and serum was pooled and diluted to create an infectious “mouse-adapted” PgV (maPgV) stock.

Stat1 mediates signaling from both the IFNα/β receptor and the IFNγ receptor as well as several additional cell-type dependent receptors. To determine if the susceptibility of *STAT1*^*-/-*^ mice to RPgV was mediated through a deficiency in IFNα/β or IFNγ signaling, we repeated the RPgV adaptation experiment in mice deficient in the IFNα/β receptor (*IFNAR*^-/-^), the IFNγ receptor (*IFNGR*^-/-^), and both types of IFN receptors (*IFNAGR*^-/-^) (**Fig 1C**). Zero of 3 WT mice supported RPgV infection, while 2/3 *STAT1*^-/-^ mice became infected with RPgV. 1/3 *IFNAR*^-/-^ mice became viremic with RPgV, while 0/3 *IFNGR*^-/-^ and 0/3 *IFNAGR*^-/-^ mice became infected, suggesting that PgV cross-species infection is restricted by type-I-IFN/Stat-1 signaling. To determine if the mice that remained RPgV-negative had developed anti-RPgV immunity that could account for their lack of viremia, we inoculated these mice with our maPgV stock which resulted in robust infection in all animals (**Fig 1C**), indicating that maPgV infection was unaffected by prior RPgV exposure.

### The kinetics of maPgV infection is highly reproducible and independent of the route and dose of infection

**Fig 2:**
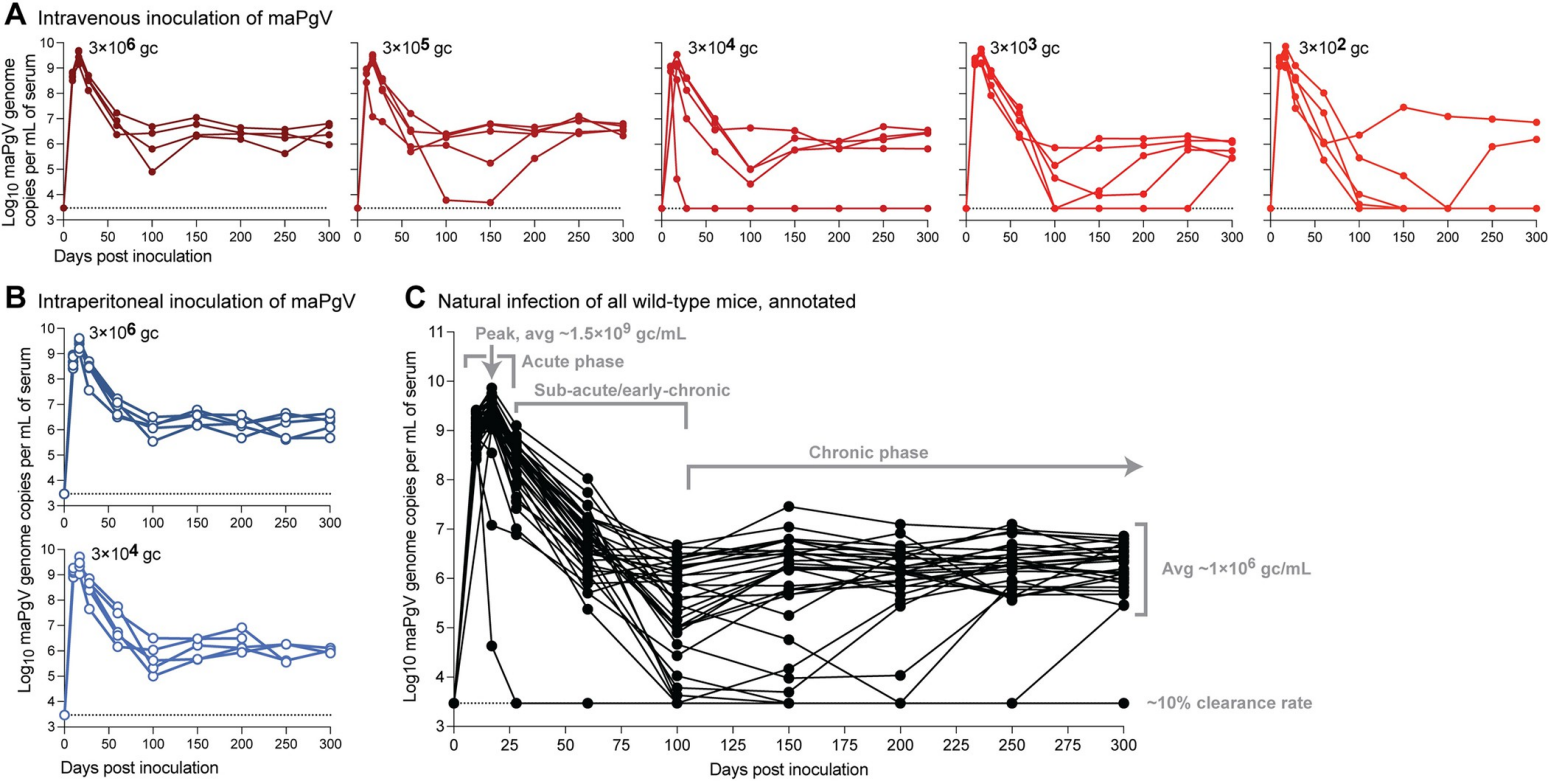
Persistent infection of wild-type mice with maPgV. Serum viral loads in wild-type mice inoculated with maPgV via (**A**) retro-orbital (i.e., intravenous) injection (red) or (**B**) intraperitoneal injection (blue), n = 5 mice per group. (**C**) Viral loads of all animals in the route × dose study, with key phases of infection annotated in gray. Limit of detection for the viral load assay is shown as a dashed line.

The route and dose of infection can have a profound impact on many aspects of viral infection including the kinetics of viral dissemination, disease, and the establishment of chronic infection versus the establishment of immune control. Thus, we examined various inoculation doses and routes to examine whether maPgV could cause persistent infection in WT mice. We inoculated mice via retro-orbital (*i*.*e*., intravenous) injection with doses of maPgV ranging across 4 orders of magnitude (3×10^6^–3×10^2^ gc) (**Fig 2A**) or 2 orders of magnitude (3×10^6^–3×10^4^ gc) via intraperitoneal injection (**Fig 2B**) and followed viral loads in these animals for 300 days. All mice became infected, with viremia detectable in all animals by 10 dpi. However, neither route nor dose had a significant impact on the trajectory of maPgV infection. Compiling data from all animals in the dose × route study shows that maPgV achieves peak viremia of ~1.5×10^9^ gc/mL at ~15–17 dpi, which gradually decreases to a set-point of ~1×10^6^ gc/mL by ~100 dpi. Approximately 10% of animals control their viremia to undetectable levels during this time, with most instances of control occurring between 100–150 dpi.

### A single mutation in the E2 envelope glycoprotein is important for initial murine adaptation of RPgV

**Fig 3:**
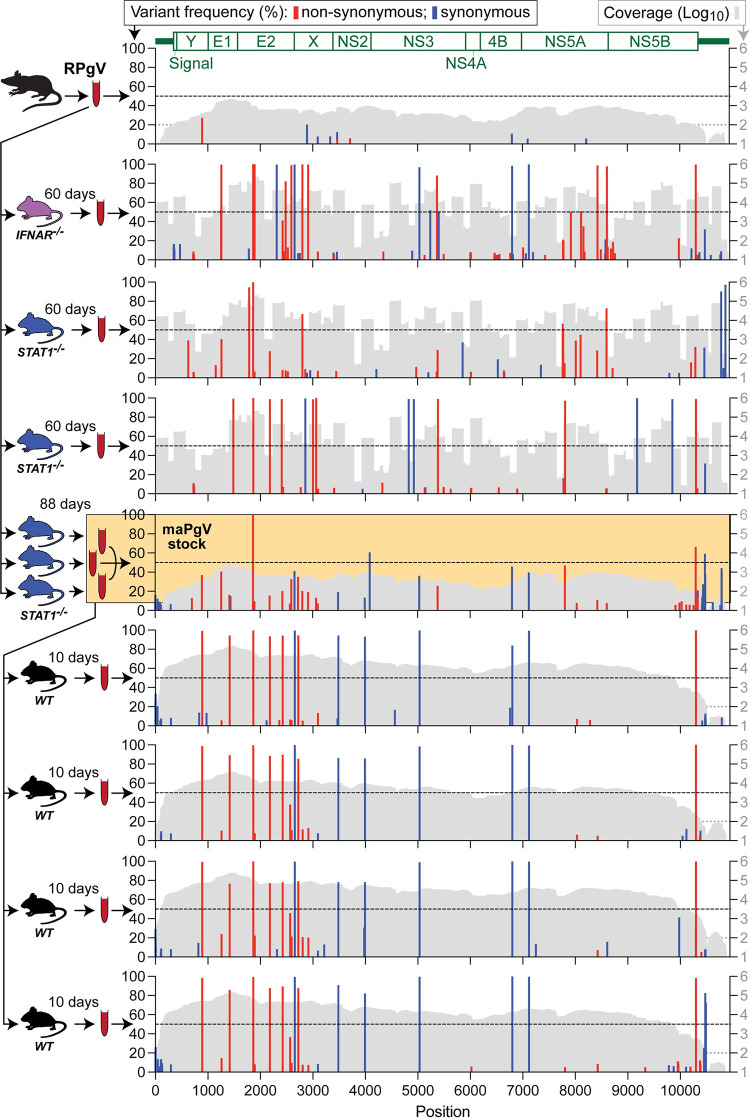
A single mutation in the E2 envelope glycoprotein (R80L) is important for murine adaptation of RPgV. Illumina deep sequencing of RPgV at various points during mouse adaptation. The genome position of RPgV/maPgV is shown along the X-axis, with a schematic of predicted mature proteins shown in green across the top. The frequency of non-synonymous mutations (red) and synonymous variants >5% relative to the RPgV consensus sequence are shown along the left Y-axis, with a dashed black line denoting 50% frequency (i.e., consensus-level variants). Coverage is shown in gray on a log10 scale along the right Y-axis with a read-depth cutoff of 100 shown as a gray dashed line, below which variants were not called. The pooled “maPgV stock” described in Fig 1 is highlighted in yellow. Note that some samples were sequenced via unbiased deep sequencing and others were sequenced by multiplexed PCR amplicon sequencing, generating the “mountainous” versus “city-scape” appearing coverage plots, respectively.

To examine the adaptive mutations that enable productive RPgV infection of mice, we performed an initial “unbiased” deep sequencing characterization of our maPgV stock. The incomplete *de novo*-assembled maPgV genome was then used as a launching point for performing 5′ and 3′ rapid amplification of cDNA ends (RACE) to generate the complete maPgV genome sequence. Using the complete maPgV genome sequence, we then used pooled primer sets to generate overlapping amplicons that yielded deep sequencing coverage of >99% of the genome for most samples. Deep sequencing of the original RPgV serum sample revealed very little intra-host diversity, with none of the subsequent mutations identified in mouse-adapted PgV descendants observable at >5% frequency (**Fig 3**). Upon inoculation of RPgV into *STAT1*^-/-^ or *IFNAR*^-/-^ mice, mutations accumulated throughout the genome. Non-synonymous mutations clustered primarily in structural genes, predominantly E2 and X, although viruses in one *STAT1*^-/-^ and one *IFNAR*^-/-^ mouse also accumulated non-synonymous mutations in NS5A and NS5B. Synonymous mutations accumulated throughout the non-structural genes, and there were few mutations that arose in the 5′ or 3′ UTR. Only one mutation––a non-synonymous mutation in the putative E2 glycoprotein (R80L)––was identified in the RPgV genome of mice initially infected with wild-type RPgV. Of note, only the pooled “maPgV stock” caused robust infection in WT mice; the other murine-adapted RPgV descendants caused low-titer viremia in WT mice that was below the threshold needed for sequencing.

### Thirteen mutations confer full murine adaptation of RPgV

**Fig 4:**
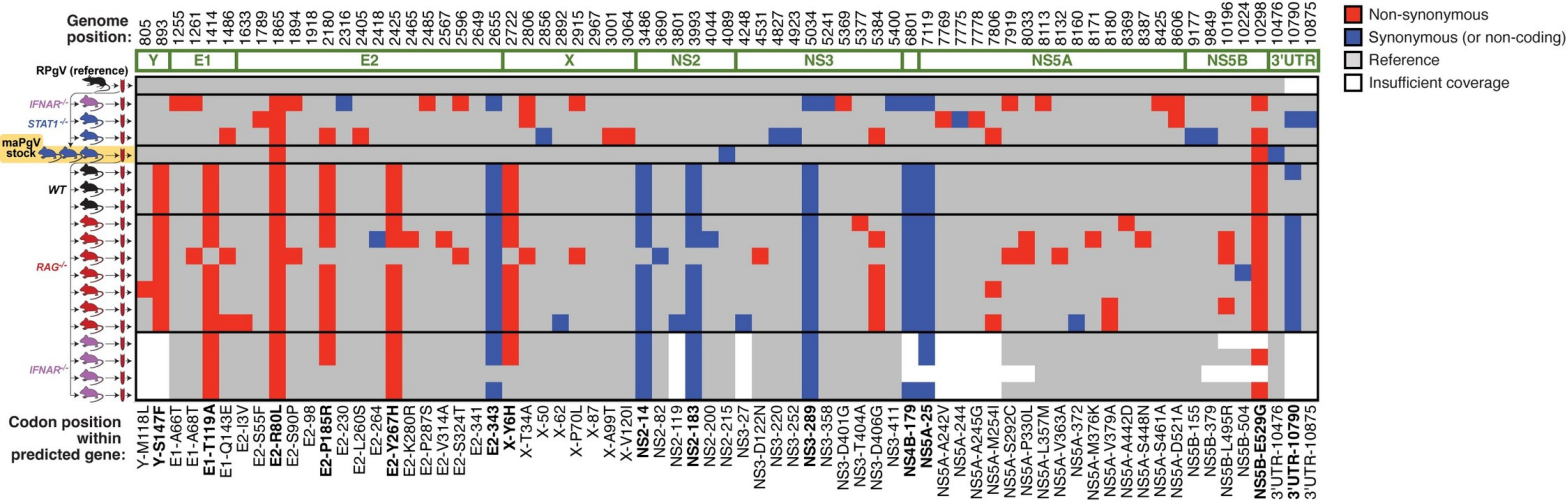
Thirteen mutations confer full murine adaptation of RPgV. Summary of all consensus-level variants detected in the expanded sequencing dataset. Equivalent analysis to that shown for a subset in Fig 3 can be found in S1 Fig. Non-synonymous and synonymous variants are shown in red and blue, respectively. Coverage > 100 reads is shown in gray. The thirteen mutations that consistently accumulate during adaptation to the murine host are in bold along the bottom.

Passage of the pooled maPgV stock into WT mice resulted in the rapid and consistent accumulation of 12 additional mutations, with non-synonymous mutations clustered in structural genes (except for one mutation in NS5B, the putative RNA-dependent RNA-polymerase) and synonymous mutations in non-structural genes (except for one silent mutation in E2) (**Figs 4 and S1 and Table 1**). The accumulation of these additional 12 single nucleotide polymorphisms (SNPs) in an immunocompetent host indicated that they might be important for evading additional aspects of the immune system. To test this hypothesis, we infected *IFNAR*^*-/-*^ and *RAG*^-/-^ mice (which lack mature T and B cells) with the maPgV stock and sequenced virus at peak viremia (15 dpi for *IFNAR*^*-/-*^ and 200 dpi for *RAG*^-/-^). Remarkably, in both groups of mice, all 13 of the fully mouse-adapting mutations rose to consensus levels without the consistent accumulation of additional mutations. Although the functional implications of these mutations remain to be determined, it seems that this additional set of 12 mutations, in addition to the single E2 mutation at R80L, is needed to confer full adaptation to the murine host.

**Table 1:** Mouse-adapting mutations in RPgV.

Genome position	RPgV nt	maPgV nt	Polymorphism Type	Codon Change	Predicted protein	AA residue	AA Change
893	C	U	SNP (transition)	UCC -> UUC	Y	147	S -> F
1414	A	G	SNP (transition)	ACU -> GCU	E1	119	T -> A
1865	G	U	SNP (transversion)	CGC -> CUC	E2	80	R -> L
2180	C	G	SNP (transversion)	CCU -> CGU	E2	185	P -> R
2425	U	C	SNP (transition)	UAC -> CAC	E2	267	Y -> H
2655	C	U	SNP (transition)	GUC -> GUU	E2	343	
2722	U	C	SNP (transition)	UAC -> CAC	X	6	Y -> H
3486	U	C	SNP (transition)	CCU -> CCC	NS2	14	
3993	C	U	SNP (transition)	UGC -> UGU	NS2	183	
5034	G	A	SNP (transition)	GUG -> GUA	NS3	289	
6801	A	G	SNP (transition)	UUA -> UUG	NS4B	179	
7119	U	C	SNP (transition)	GGU -> GGC	NS5A	25	
10298	A	G	SNP (transition)	GAG -> GGG	NS5B	529	E -> G
10790	-	C	Insertion		3′UTR		

### Structural implications of non-synonymous mutations in the PgV envelope glycoproteins

**Fig 5:**
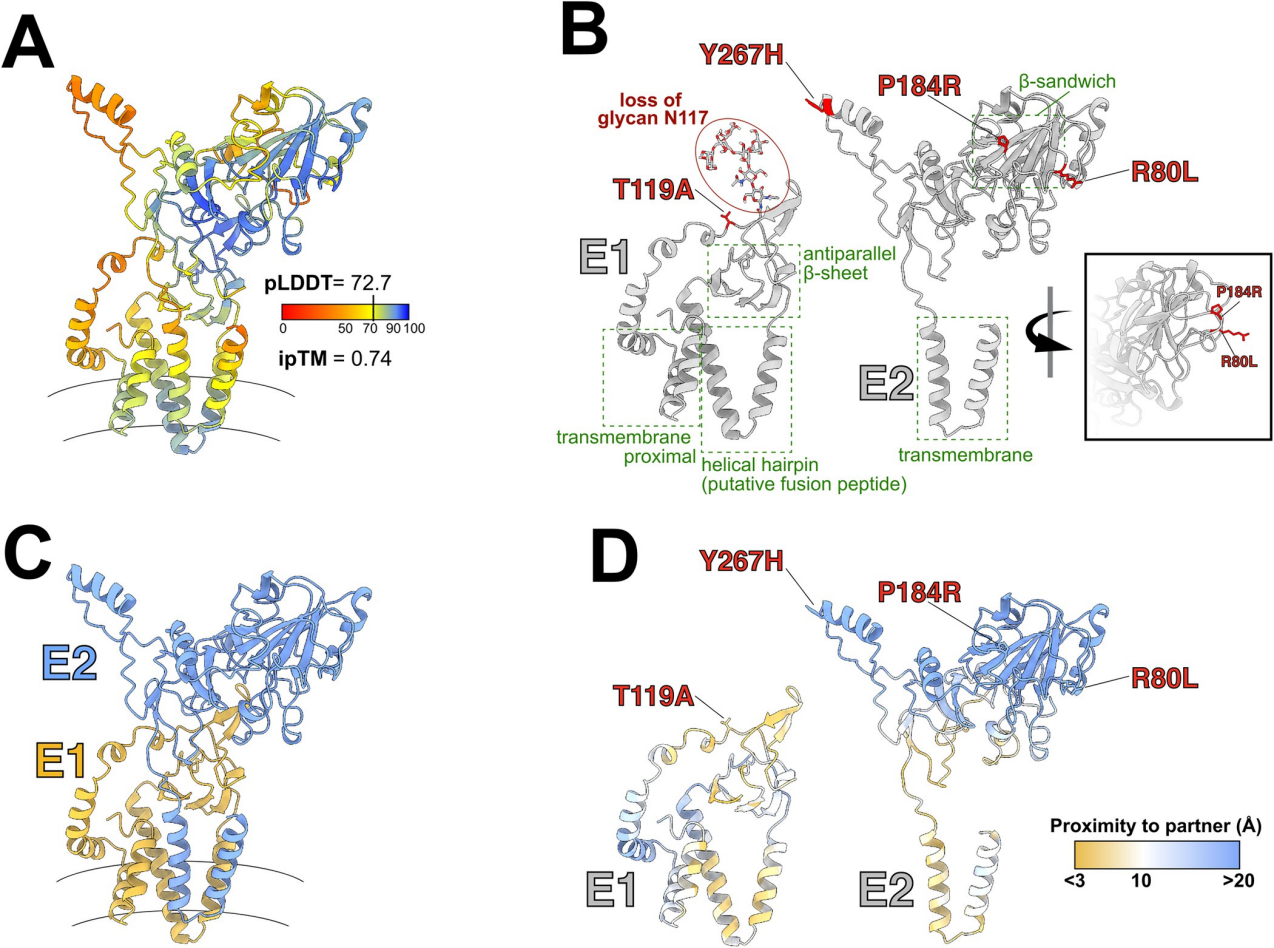
Predicted E1/E2 glycoprotein structures and mouse-adapting mutations. (**A**) ColabFold-AlphaFold2 prediction of the E1/E2 heterodimeric envelope glycoprotein complex for RPgV. Color scale indicates the confidence of the prediction (predicted local distance differences test [pLDDT] red = low confidence; blue = high confidence). Interface predicted template modeling (ipTM) score provides a metric of the quality of heterodimer modeling, values closer to one are higher confidence. Curved lines indicate the approximate location of the viral membrane. (**B**) Annotations of the separated E1 and E2 glycoproteins, with previously described features common to hepaciviruses and pegiviruses highlighted in green and the observed mouse-adapting mutations shown in red. Inset displays the outward facing location of P184R and R80L on E2 (**C**) E1/E2 complex highlighting each subunit (E1:gold; E2: blue). (**D**) E1-E2 interaction interface, residues are color coded by their shortest distance to the partner protein (C⍺ to C⍺ distance [Å], as shown in the key) with gold denoting areas of contact and blue denoting those that are distant.

Four of the seven non-synonymous mutations that we identified in fully-mouse-adapted PgV occurred in the regions that putatively encode the envelope glycoproteins, E1 and E2. These glycoproteins are predicted to form a heterodimer on the virion surface, similar to what has been shown for phylogenetically-related hepaciviruses and pestiviruses [13–17]. To place the non-synonymous mutations in a structural context we first modeled the E1/E2 complex using ColabFold-AlphaFold2 (**Fig 5A** [18,19]). Consistent with our modeling of other pegivirus glycoproteins, we found highly-conserved structural features within E1 (a central antiparallel beta-sheet, a helical hairpin analogous to the putative fusion peptide of HCV, and a helical transmembrane proximal region) and E2 (a beta-sandwich and transmembrane helical hairpin) (**Fig 5B**). Overlaying the mouse-adapting mutations onto these structures revealed two types of mutations. The three mutations in E2 (Y267H, P184R, and R80L) occur within the distal portion of the glycoprotein complex that presumably mediate interactions with host factors and/or antibodies. In particular, P184R and R80L are closely juxtaposed on outwardly facing loops that extend from the central beta-sandwich. In contrast, the single mutation in E1 (T119A) occurs within the predicted E1-E2 interface (**Fig 5C** and **5D**). Furthermore, E1E2 are heavily glycosylated (**S2 Fig**), and this mutation ablates the glycosylation signal at E1:N117. These changes may alter the molecular interaction between E1 and E2, which may in turn modulate their fusion activity. However, ColabFold-AlphaFold2 models generated from the maPgV E1E2 sequences were very similar to RPgV E1E2 models with only some positional changes in short flexible regions and with negligible impact on the pLDDT and ipTM metrics (**S3 Fig**). We, therefore, may expect the mutations exert only subtle effects that ‘fine tune’ E1E2 to the murine host.

### Structural implications of synonymous mutations on PgV RNA structure

**Fig 6:**
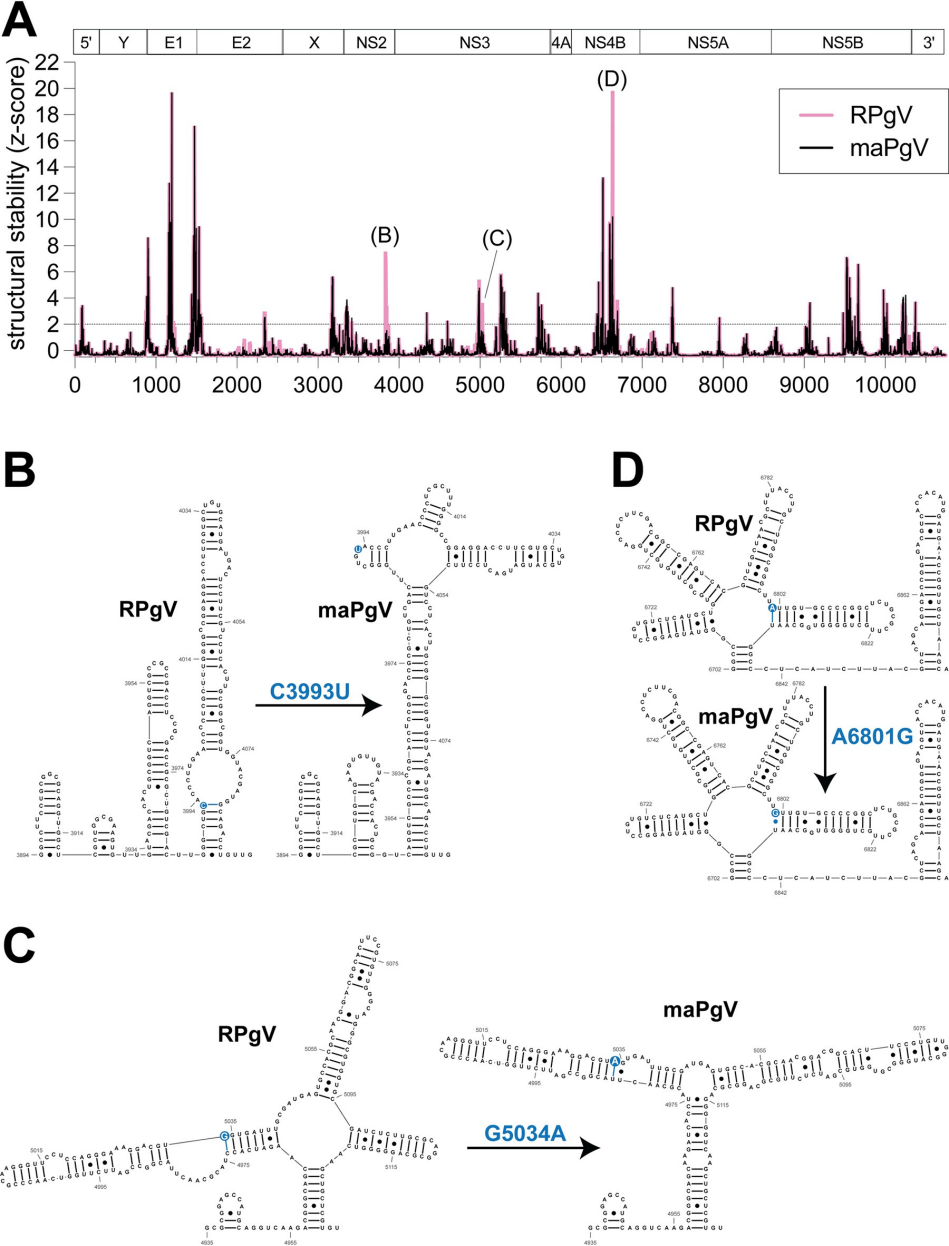
Predicted RPgV RNA genome structure and mouse-adapting mutations. (**A**) RNAfold analysis comparing RPgV (pink) to maPgV (black) across the entire PgV genome, with genomic architecture depicted above the graph. Predicted RNA secondary structure of 200 nucleotide windows (step size = 1 nt) were computed using RNAfold and the RNA structure score (RSS; frequency of the MFE/ensemble diversity) and z-score of the RSS were calculated. Regions where structural stability differs significantly between RPgV and maPgV are identified and RNA structures are shown in greater detail, with the SNP highlighted in blue (**B-D**).

To investigate the impact of the mouse-adapting mutations on the secondary structure of RPgV RNA, we used RNAfold [20] to perform a sliding window analysis and generate RNA structure scores (RSS) [21] of the RPgV and maPgV genomes (**Fig 6A**). Comparison of the predicted RNA secondary structure of RPgV and maPgV revealed the presence of several regions of conserved structure between both viruses (e.g. E1 and NS5B). Notably, of the six synonymous SNPs identified in maPgV, three of these (C3993T, G5034A, and A6801G) were found to alter the predicted secondary structure of maPgV as compared to RPgV, and were specifically found to reduce the predicted thermodynamic stability of RNA structures in maPgV compared to RPgV. These mutations imparted various changes to the predicted local base-pairing, with C3993T exerting a modest change to the secondary structure (**Fig 6B**), G5034A exerting minimal change (**Fig 6C**), and A6801G prompting a dramatic reorganization of the local secondary structure (**Fig 6D**). Although the functional impact(s) of these mutations remains unknown, this finding could explain the selection of at least some of the synonymous mutations that were observed in the fully mouse-adapted PgV.

### Immune determinants of PgV persistence

**Fig 7:**
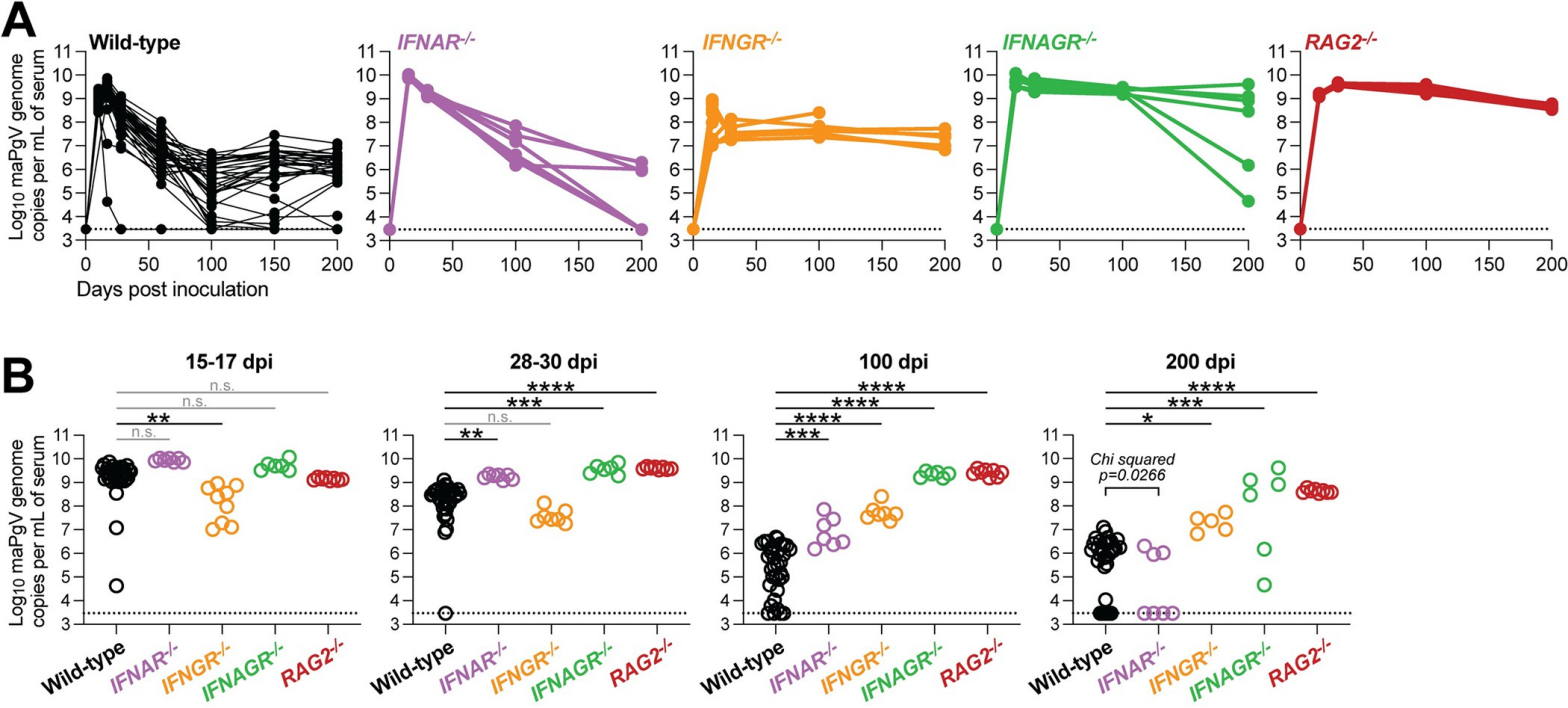
Type-I interferon signaling contributes to PgV persistence. (**A**) Serum viral loads of maPgV in various immunocompromised mouse strains over time (wild-type: black, n = 25; *IFNAR*^-/-^: purple, n = 7; *IFNGR*^-/-^: orange, n = 7; *IFNAGR*^-/-^: green, n = 6; *RAG*^-/-^: red, n = 8). Combined data from two independent cohorts are shown. (**B**) Comparison of maPgV serum viral loads in immunocompromised mice strains at various time points during infection using one-way ANOVA in relation to wild-type mice, corrected for multiple comparisons (*:p≤0.05; **:p≤0.01; ***:p≤0.001; ****:p≤0.0001). Note: data from Wild-type mice is from the same data-set shown in Fig 2; data from immunocompromised mice is combined from two independent cohorts.

PgV persistence remains a poorly understood phenomenon, without a clear understanding of how various components of the immune system impact the level and duration of PgV viremia. To investigate this, we first examined the role of type-I interferon (IFN)––a master regulator of antiviral immunity––on maPgV infection by infecting mice deficient in the IFNα receptor (*IFNAR*^-/-^). Compared to maPgV viremia in WT mice, *IFNAR*^-/-^ mice displayed a similar peak of viremia but maintained higher viral loads in the sub-acute/early-chronic phase of infection (**Fig 7A and 7B**). Interestingly however, a majority of *IFNAR*^-/-^ mice (~60%) went on to clear maPgV infection by 200 dpi, compared to a clearance rate in WT mice of only ~10%, suggesting that intact type-I IFN signaling in late-chronic PgV infection can enable PgV persistence. IFNγ can also have a significant impact on chronic viral infection, and so we examined the effect of IFNγ on maPgV infection by infecting mice deficient in the IFNγ receptor (*IFNGR*^-/-^), along with mice deficient in both IFNα and γ receptors (*IFNAGR*^-/-^). In contrast to *IFNAR*^-/-^ mice, *IFNGR*^-/-^ mice displayed a blunted acute-phase maPgV peak followed by a slightly elevated chronic-phase set point without any instances of viral clearance. *IFNAGR*^-/-^ mice had an acute-phase maPgV trajectory similar to *IFNAR*^-/-^ mice, but animals diverged into one of two patterns in chronic phase, either sustaining very high titers or exhibiting declining viral loads, potentially an early indicator of eventual viral clearance similar to what was observed in *IFNAR*^-/-^ mice. We also examined the impact of adaptive immunity on maPgV infection by infecting mice deficient in T and B cells (*RAG*^-/-^): these mice consistently sustained high levels of maPgV viremia.

### PgV does not require PD-1-mediated immune tolerance for persistence

**Fig 8:**
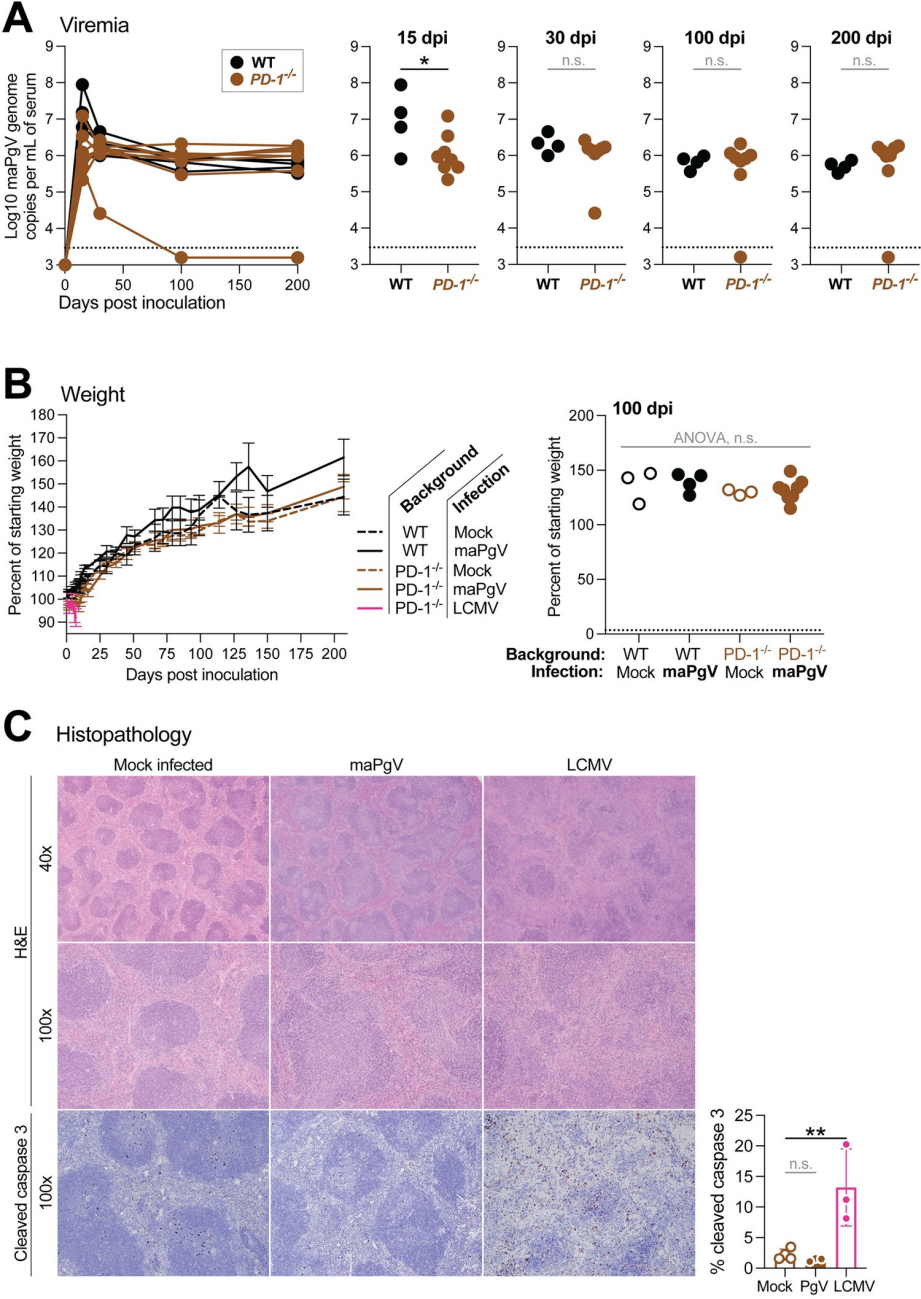
PgVs do not require PD-1-mediated immune tolerance for persistence.

The induction of immune tolerance plays a well-described role in the establishment and maintenance of persistence in several viral infections [22]. In particular, upregulation of programmed cell death protein-1 (PD-1), which inhibits the activation of virus-specific T lymphocytes (among other, less well-described functions), plays a central role in the induction of tolerance, blunting the anti-viral immune response to prevent immunopathology at the cost of persistence [23,24]. Indeed, mice deficient in the PD-1/PD-L1 signaling axis succumb to fatal immunopathology when infected with lymphocytic choriomeningitis virus (LCMV, clone 13), a commonly used model of RNA virus persistence [25]. To determine if induction of PD-1 mediates PgV persistence––without any way of measuring PgV-specific T cells––we infected *PD1*^*-/-*^ mice with maPgV. *PD1*^*-/-*^ mice exhibited slightly lower acute-phase (15 dpi) viral loads compared to WT mice, but this difference disappeared as infections progressed (**Fig 8A**). *PD1*^*-/-*^ maPgV-infected mice also displayed no signs of disease, normal splenic architecture, normal levels of apoptosis in the spleen, and normal weight gain relative to age/sex-matched naive *PD1*^*-/-*^ mice, naive WT mice, or maPgV-infected WT mice (**Fig 8B and 8C**). In contrast, *PD1*^*-/-*^ mice infected with LCMV (clone 13) displayed effaced splenic architecture with apoptotic germinal centers (as determined by cleaved caspase 3 staining) and rapid weight loss, as expected [25].

(**A**) Serum viral loads of maPgV in wild-type (black, n = 4) versus *PD-1*^-/-^: (brown, n = 8) with unpaired t-test used to determine significance (*:p≤0.05). (**B**) Weight gain/loss data for wild-type (black) and *PD-1*^-/-^ (brown) mice inoculated with mock (dashed line or open circle), maPgV (solid line or solid circle), or LCMV (pink); ANOVA at 100 dpi with correction for multiple comparisons. (**C**) Hematoxylin and eosin staining (top) or immunohistochemistry for the apoptotic marker cleaved caspase 3 (bottom) of splenic tissue from *PD-1*^-/-^ mice infected with mock, maPgV (106 dpi), or LCMV (7 dpi). Cleaved caspase 3 staining was digitally quantified and compared using ANOVA with correction for multiple comparisons, with the Mock group serving as the reference group (**:p≤0.005), error bars show SD.

### Natural PgV immunity can be achieved via multiple immune mechanisms

**Fig 9:**
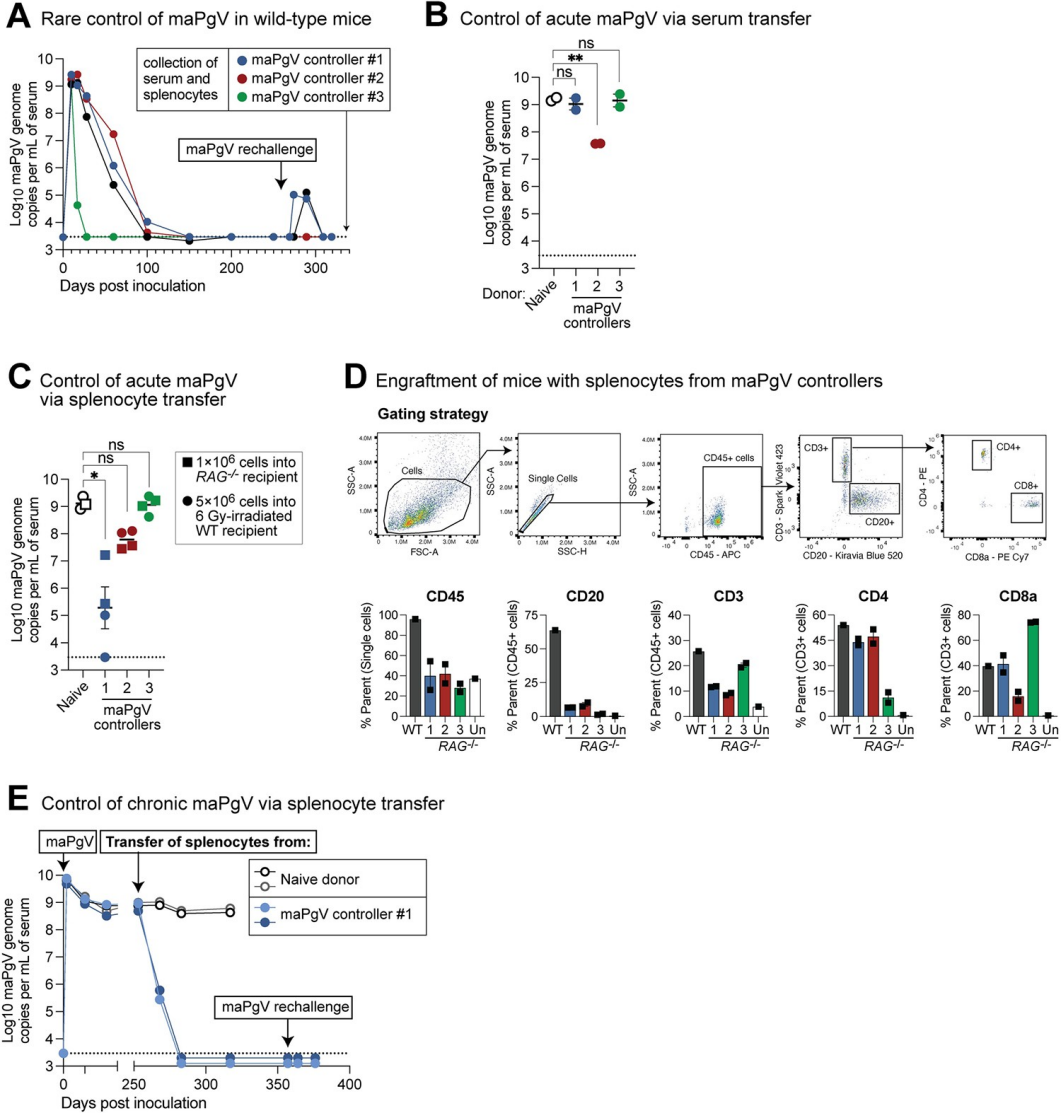
Natural PgV immunity can be achieved via multiple immune mechanisms. (**A**) Trajectories of viremia in 4 mice that ultimately cleared maPgV infection and were resistant to rechallenge. (**B**) Transfer of serum (100μL, via retro-orbital injection) from the maPgV-immune donors in part “A” into wild-type mice two days prior to maPgV inoculation. Colors correspond to the donors from part “A” throughout the figure. Viral loads are from 15 dpi (i.e., “peak”) maPgV viremia; one-way ANOVA with correction for multiple comparisons (**:p≤0.01). (**C**) Transfer of splenocytes from donors in part “A” into sublethally-irradiated (6 Gy) wild-type mice (5×10^6^ cells via retro-orbital injection, circular symbols) or *RAG*^*-/-*^ mice four days prior to maPgV inoculation. Viral loads are from 15 dpi (i.e., “peak”) maPgV viremia; one-way ANOVA with correction for multiple comparisons (*:p≤0.05). (**D**) Flow cytometry plots demonstrating engraftment of donor lymphocytes in the maPgV-infected *RAG*^*-/-*^ mice from part “C.” (**E**) Transfer of cryopreserved splenocytes from donor #1 (see part “A”) into chronically-infected *RAG*^-/-^ mice (closed circles), with transfer of splenocytes from a naive donor (open circles) serving as a control.

Approximately 10% of mice infected with maPgV control viremia to undetectable levels by 200 dpi. Most of these animals achieve undetectable viremia between 100–150 dpi, but we also observed one example of rapid maPgV clearance immediately following a typical acute-phase peak. To examine correlates of immunity in these relatively rare “controllers,” we first re-challenged these animals with maPgV, which led to spikes in viremia in 2/4 animals that eventually cleared (**Fig 9A**). We then harvested splenocytes and serum from these controllers and transferred these tissues into PgV-naive mice to examine correlates of PgV immunity. Transfer of serum from maPgV-immune donors into wild-type mice (100μL via retro-orbital injection) two days prior to maPgV inoculation had no effect for 2/3 donors, but serum from one donor (i.e., donor #2) resulted in a ~100-fold reduction in peak maPgV viremia at 15 dpi (**Fig 9B**). Transfer of splenocytes into sublethally-irradiated wild-type mice (5×10^6^ cells via retro-orbital injection) four days prior to maPgV infection had no effect for 2/3 donors, but splenocytes from one donor (i.e., donor #1) resulted in a substantial reduction in peak maPgV viremia at 15 dpi, with one recipient having undetectable viral loads (**Fig 9C**). To ensure that the lack of maPgV immunity in donors #2 and #3 was not due to poor engraftment, we also repeated this transfer experiment in *RAG-/-* mice (1×10^6^ cells via retro-orbital injection). All *RAG-/-* recipients had detectable donor immune cells at 15 dpi, but again mice that received splenocytes from donor #1 had significantly reduced maPgV viral loads (**Fig 9C and 9D**). To determine if the anti-PgV immunity mediated by the splenocytes of donor #1 could control maPgV infection in chronically-infected RAG-/- mice, we adoptively transferred cryopreserved splenocytes from this donor into *RAG*^*-/-*^ mice that had been infected for >250 days. Within 15 days post-transfer, maPgV viral loads had decreased by three orders of magnitude, from a set-point of ~1×10^8−9^ gc/mL to ~1×10^5−6^ gc/mL (**Fig 9E**). In contrast, transfer of splenocytes from a maPgV-naive donor had no impact on chronic phase viral loads. By 20 days post-transfer, maPgV viremia was undetectable in *RAG*^*-/-*^ mice that had received anti-maPgV splenocytes. These mice remained aviremic, even after maPgV rechallenge.

## Discussion

The laboratory mouse has become the dominant animal model for studies in virology and immunology for a multitude of reasons including cost, size, housing requirements, reproductive time, robust genetics, transgenic technologies, and abundant reagents. However, there are few mouse models of chronic viremia and these often require the use of an immunocompromised host for persistent viral replication [26]. Additionally, no immunocompetent mouse models exist for studying infections by viruses in the related *pegi*-, *hepaci*-, or *pesti- virus* genera; indeed, a mouse-adapted rat hepacivirus is unable to cause persistent viremia in immunocompetent mice [27,28]. Accordingly, our creation of a mouse-adapted pegivirus (maPgV) represents a significant advance for the field, as it allows for highly-reproducible persistent infection in wild-type mice, allowing for further exploration of maPgV as a *bona fide* model of chronic infection. The close phylogenetic relationship between maPgV and HPgV, and their similar course of infection in mice and humans, respectively, further emphasizes the utility of the maPgV given that many viruses used as chronic infection models in mice do not have a directly-relevant human counterpart.

Our infection of mice with RPgV, but not HPgV, adds to a growing body of evidence indicating that PgVs have a narrowly-restricted species-tropism [11,12]. Although the molecular interactions governing PgV species-tropism remain unknown, the unique susceptibility of innate-immune-compromised (*STAT1*^-/-^ and *IFNAR*^-/-^) mice to RPgV suggests that innate immune effectors contribute to restrict cross-species PgV infection. Thus, we propose that RPgV adaptation to the murine host occurred in two steps: first, in the absence of innate immune restriction, RPgV was able to establish an inefficient, low-level infection in mice. This then allowed RPgV to acquire additional mutations that were selected for enhanced/efficient interactions with the murine host, many of which reside in exposed surfaces of the envelope glycoprotein complex. Future mutation-function studies comparing the RPgV and maPgV genomes will help to elucidate the interactions between these viruses and their respective hosts; however, attempts to rescue an infectious maPgV clone have so far been unsuccessful.

The immune response to PgVs has often been conceptualized as weak or even non-existent, as evidenced by the ability of PgVs to persistently infect immunocompetent hosts without accumulating mutations indicative of adaptive immune pressure/escape [12,29,30]. Nevertheless, *RAG*^-/-^ mice sustain chronic-phase levels of replication that are ~1000-fold higher than those of WT mice, indicating that the adaptive immune system plays a significant role in restricting viral replication to enable the establishment of the chronic-phase set-point. Determining why this relative degree of adaptive immune control takes so long (~100 days) to develop, and why further control beyond the set-point is not possible in most WT animals, will likely require the development of currently non-existing PgV-specific immunologic reagents (*e*.*g*., antibodies, MHC:peptide tetramers). However, our studies in IFNAR-deficient mice (IFNAR^-/-^ and IFNAGR^-/-^) provide some initial clues. Specifically, elevated type-I IFN signaling is known to interfere with effective T cell response in chronic viral infections [31,32], and the high rate of maPgV clearance in *IFNAR*^-/-^ mice suggests that type-I IFN-induced T cell dysfunction may also be playing a role in PgV persistence. This could explain the higher prevalence of HPgV infection in people with cancer and HIV infection, as chronic elevation of type-I IFN signaling is observed in both of these conditions [31]. However, whether PgV is causing elevated type-I IFN signaling or merely benefiting from its presence remains unknown and, in any event, does not explain the positive impact of PgV infection on HIV disease progression.

Similarly, the determinants of PgV clearance––which we demonstrate to be a distinct (and rare) phenomenon compared to the control of PgV viremia to a chronic phase set-point (which occurs in nearly all wild-type animals)––remains elusive. However, our adoptive transfer studies on these “elite PgV controllers” suggests that PgV immunity can but can likely be achieved via multiple immune mechanisms. Although persistent viremia is a unique feature of this mouse model among models of persistent RNA virus infection in mice, the low rate of PgV clearance certainly complicates the study of anti-PgV immunity through classical techniques (e.g., immune cell depletion) that rely upon the conferment of persistence to an otherwise non-persisting viral infection [26,28].

Interestingly, our data indicates that PD-1/PD-L1 signaling contributes minimally to PgV persistence, despite the importance of this pathway in mediating the persistence of many viral infections [24,33,22]. This is perhaps reassuring given the prevalence of HPgV and the rising frequency of PD-1 “checkpoint inhibitor” therapies for treating cancer. But more broadly, this finding implies that PgVs employ a highly novel mechanism of immune evasion that is independent of the PD-1/PD-L1 tolerance axis that is a hallmark of many chronic viral infections.

## Methods

### Ethics statement

All experiments were conducted by trained personnel and in compliance with Washington University in St. Louis and the University of Wisconsin–Madison policies and procedures. All animal studies were approved by the IACUCs at these respective institutions prior to initiation of experiments.

### Discovery of RPgV and passage history in rats

RPgV was isolated from a serum sample of a female brown rat (*Rattus norvegicus*) captured in the city of New Orleans, USA. The serum sample was used to infect outbred Sprague Dawley rats, and one of these rats (no. 398) was euthanized at 14 dpi to confirm infection and generate a virus stock. This virus stock was used to infect inbred Lewis lab rats. One Lewis rat (no. 971) was euthanized on day 11, and this was used as stock for mouse adaptation studies.

### Historical perspective on adaptation experiments

Initial studies adapting RPgV to infect mice were conducted at Washington University in St. Louis in 2018–2019 (Fig 1A and 1B); however, these were interrupted by the COVID-19 pandemic. All subsequent studies were performed at the University of Wisconsin–Madison between 2021 and 2024.

### Mice

All mice were obtained from Jackson Laboratories (Bar Harbor, ME) and bred in the Mouse Breeding Core at UW–Madison (see Table 2 for details). Note, both Rag1 and Rag2 knockout mice were used throughout the study out of convenience. Rag1 and Rag2 knockout mice have essentially the same phenotype, and a knockout of either gene suffices to eliminate the adaptive immune system. Thus, for simplicity and accuracy, we have used the term “Rag” throughout the study.

**Table 2:** Transgenic mice used in this study.

Abbr.	Technical name	Jax ID
WT	c57BL6/J	000664
STAT1^-/-^	B6.129S(Cg)-Stat1^tm1Dlv^/J	012606
IFNAR^-/-^	B6(Cg)-Ifnar1^tm1.2Ees^/J	028288
IFNGR^-/-^	B6.129S7-Ifngr1^tm1Agt^/J	003288
IFNAGR^-/-^	B6.Cg-Ifngr1^tm1Agt^Ifnar1^tm1.2Ees^/J	029098
PD1^-/-^	B6.Cg-Pdcd1^tm1.1Shr^/J	028276
RAG^-/-^	B6.129S7-Rag1^tm1Mom^/J	002216
	B6.Cg-Rag2^tm1.1Cgn^/J	008449

### 5′ RACE

The SMARTer RACE 5’/3’ kit (Takara Bio, USA) was used to generate cDNA according to the manufacturer’s instructions using a maPgV-specific reverse primer (TGCGAGAGCCGTCAGCCACA). PCR amplification was then performed with the kit’s universal primer mix and a second maPgV-specific reverse primer (CCGTAGCAGGCGGGTCAGCA). Finally, nested PCR was performed using the SMARTer RACE 5’/3’ kit’s short universal primer and a third maPgV-specific reverse primer (CGCGCAAGCCCTTCTGGATA), yielding a product of ~600bp. This PCR product was submitted for Sanger sequencing (UW Biotechnology Center) using a fourth maPgV-specific reverse primer (TCCGGCGTGGTTGTTGTGTTT), yielding high-quality chromatograms that clearly identified the “template-switching oligo” indicative of the 5′ end of the maPgV genome.

### 3′ RACE

Extracted maPgV RNA was subjected to poly(U) tailing using a poly-U polymerase (NEB, Ipswitch, MA) followed by cDNA synthesis with Superscript IV RT (Invitrogen, Waltham, MA) and a poly-A primer (GAATCGAGCACCAGTTACGCATGCCGAAAAAAAAAAAAAAAAAAAAAMN). PCR amplification was then performed using the Platinum SuperFi II DNA polymerase and a maPgV-specific forward primer (GGGGTTGGCCAGCCGATTGT) with a reverse primer complementary to the sequence added during cDNA synthesis (GAATCGAGCACCAGTTACG). This product was then subjected to nested PCR using a second maPgV-specific forward primer (CCGGCTCGGTTCAGCCATCC) and a third reverse primer (GAGCACCAGTTACGCATGCC). The nested PCR product was then subjected to Oxford Nanopore sequencing (Plasmidsaurus, Eugene, OR) which revealed homology to the known maPgV 3′ genome extending into a poly-T tract indicative of the distal 3′ end of the genome initially labeled by the poly-U polymerase.

### Structural modeling

Structures for the RPgV and maPgV E1E2 complex were predicted using the ColabFold implementation of AlphaFold2 [18,19] onGoogle Colab Cloud Computing. Calculation of per-residue inter-chain distances were calculated using a custom python script. Glycan modeling was performed using CHARM-GUI [34] and AlphaFold3 [35]. Molecular modeling and visualization was performed using UCSF ChimeraX [36].

### LCMV

Lymphocytic choriomeningitis virus (Clone 13) stocks were grown in BHK-21 cells and viral titers were determined by focus-forming assay on Vero cells as previously described [37]. *PD1*^-/-^ mice aged 7–11 weeks were inoculated intravenously with 2x10^6^ focus forming units diluted in sterile PBS.

#### RNA extraction

For serum viral loads, RNA was extracted from 20μL of serum using the KingFisher Flex (ThermoFisher, Waltham, MA) with MagMax reagents. Carrier RNA was omitted for samples destined for RACE or sequence-independent single-primer amplification (SISPA) sequencing but not for samples destined for RT-qPCR or Primal sequencing.

#### RT-qPCR for PgV RNA

Extracted RNA was subjected to RT-qPCR using the TaqMan RNA-to-CT 1-Step Kit (ThermoFisher, Waltham, MA) in a 20μL reaction with 0.5μM of primers and 0.1μM of probe labeled with FAM and ZEN/Iowa Black quenchers (IDT, Coralville, IA). Primer/probe sets for RPgV/maPgV were as follows: Forward: ATCACGGGTAAGCTGGTTTG; Reverse: GGAAACCAAGCAGAGTGAGC; Probe: CGGACACTTCCCAGTCTGT. Primer/probe sets for HPgV were as follows: Forward: GGCGACCGGCCAAAA; Reverse: CTTAAGACCCACCTATAGTGGCTACC; Probe: TGACCGGGATTTACGACCTACCAACCCT. Thermocycling was performed on a Quantstudio 6 Pro (Applied Biosystems, Waltham, MA) with a 96-well block (0.2mL) under the following conditions: 48°C for 15 min followed by 95°C for 10 min, then 50 cycles of 95°C for 15 sec followed by 60°C for 1 min. A RNA standard was made by cloning a fragment of the maPgV genome sequence into the pJET1.2/blunt vector (Invitrogen, Waltham, MA). After linearization of the construct, transcription was performed in vitro for 6 h with the MEGAscript T7 transcription kit (Invitrogen, Waltham, MA), followed by purification using the MEGAclear transcription cleanup kit (Invitrogen, Waltham, MA), quantification, and dilution to a concentration of 1×10^10^ transcript copies per μL. Ten-fold dilutions of this transcript were used as a standard curve, which was linear over 8 orders of magnitude and sensitive down to 10 copies of RNA transcript per reaction.

#### Unbiased deep sequencing

cDNA was generated from extracted RNA using a revised SISPA approach. First, 30 μL of extracted total nucleic acids were treated with TURBO DNase (Thermo Fisher Scientific) and concentrated to 10 μL with an RNA Clean & Concentrator-5 kit (Zymo Research, Irvine, CA, USA). Next, 1 μL of Primer A (40 pmol/μL; 5′-GTTTCCCACTGGAGGATA-(N9)-3′) was added to 4 μL of concentrated viral RNA and heated in a thermocycler at 65°C for 5 min and cooled at 4°C for 5 min. Reverse transcription was performed by adding 5 μL of Superscript IV (SSIV) reverse transcription master mix containing 1 μL of deoxyribonucleotide triphosphate (dNTP; 10 mM), 0.5 μL of dithiothreitol (DTT; 0.1 M), 1 μL of PCR water, 2 μL of 5X RT buffer, and 0.5 μL of SSIV RT to the reaction mix. The mix was incubated in a thermocycler at 42°C for 10 min. Second-strand cDNA synthesis was performed by adding 5 μL of Sequenase reaction mix (3.85 μL of PCR water, 1 μL of 5X Sequenase reaction buffer, and 0.15 μL of Sequence enzyme) to the reaction mix and incubating at 37°C for 8 min. After incubation, 0.45 μL of the Sequenase dilution buffer and 0.15 μL of Sequenase were added to the reaction mix and incubated at 37°C for 8 min. To amplify the cDNA, 5 μL of the cDNA was added to 45 μL of the Primer B reaction mix containing 0.5 μL of AccuTaq LA DNA polymerase, 5 μL of AccuTaq LA 10x buffer, 1 μL of Primer B (100 pmol/μL; 5′-GTT TCC CAC TGG AGG ATA -3′), 2.5 μL of dNTP (10 mM), 1 μL of dimethyl sulfoxide (DMSO), and 35 μL of PCR water. The cDNA was amplified using the following thermocycler conditions: 98°C for 30 s, 30 cycles (94°C for 15 s, 50°C for 20 s, and 68°C for 2 min), and 68°C for 10 min. After the incubation, the amplified PCR product was purified using AMPure XP beads (Beckman Coulter, Brea, CA, USA) at a 1:1 concentration and eluted in 50 μL of PCR water. The purified PCR products were quantified with the Qubit dsDNA high-sensitivity kit (Invitrogen, Waltham, MA, USA). SISPA-prepared cDNA material was submitted to the University of Wisconsin–Madison Biotechnology Center. Samples were prepared according to the QIAGEN FX DNA Library Preparation Kit (QIAGEN, Germantown, MD, USA). The quality and quantity of the finished libraries were assessed using a Tapestation (Agilent, Santa Clara, CA, USA) and a Qubit dsDNA HS Assay Kit, respectively. Paired-end 150-bp sequencing was performed using the NovaSeq6000 system (Illumina, San Diego, CA, USA).

#### Multiplexed PCR amplicon sequencing

Primers were designed to generate overlapping amplicons of ~250bp spanning the entire maPgV genome using PrimalScheme in high-GC mode [38]. 0F:CTGTCCCTACGGTCAACTGC; 1F:TGGTGAAGGGGTTAGGGTGG; 2F:TACTGCCTGATAGGGTGCCG; 3F:TCTCTGGCTTCGGTAAGTCCC; 4F:ACATCACCGCCACTCACCA; 5F:TGGTTTGTCTTTAGTTTCTTGGTTC; 6F:GCAGAGTAGTAGGGCTTGCAG; 7F:TGGTGGCGTCTCTGTCAGG; 8F:GTTCCGGACCTCGTGTGC; 9F:CTGGGTCCAGGTCATCCTCC; 10F:GCGCAACTTTAACTCTAGCTACG; 11F:GGTGGTGGCAACACAACA; 12F:GCGGCCTGACTTTTGTGG; 13F:GCGGTTGGCGTCTAAGTACC; 14F:TCTTTGCCGTGGTCGCCT; 15F:ACGCAGGTGTTCGGAGGT; 16F:GCAACTACTATTTGTGCTGGACCT; 17F:TCTCTGGGTGACTTGGCAGG; 18F:TTTTTCTCAACGTTTAGGTTCCGC; 19F:CCGTTGCGTGGTGGTTGT; 20F:CCTGGCCGCGTACAAGATTT; 21F:GCTCTCTTCCTTTGCGCTCTT; 22F:ACCACTTGGGCTGCCGT; 23F:GCTGGCGAAGTGTTGTACGA; 24F:GGTGCTTCTGTCTTGATCGGG; 25F:CAGATTGCTTCATTGCGCACAG; 26F:CGTGTGGGTGCAACTCGTC; 27F:GGTACTGTGTGACTGTGGGC; 28F:TACATTGCTCCGACGGGGT; 29F:GGAGCCATGCAGGTCAAGAC; 30F:GGGTCAAGCTGCTCGTGTTC; 31F:GGTGGAGTGTCAACGTGTGG; 32F:TGGTGCAGGAGGATGTGGAC; 33F:TTCGTCCGTTGCGTGGG; 34F:GCAGAGGTGACACAAGAGGC; 35F:ACTACCTCACACCCTTTAGCTTTG; 36F:TGGCGCTCCAGTCTATCCAG; 37F:AGAGGGAGCTCAGCGGTTT; 38F:TCGCTGGCCTCGTCTGT; 39F:TCGATATGATCCTCGGGGTGG; 40F:GTCACCACATGGATGAACCGG; 41F:ACGTGGGTCGGACGACT; 42F:GCCGATCGGTGACCTTGAC; 43F:CATGTGGTTAGGCTGGTGGG; 44F:CAGTCACCTACAACGGGCTG; 45F:TCAAAGCTGCTGACGCGG; 46F:CGGAGGTGCTTGAGTGGCT; 47F:GTCTTAGTGCGGAGCCCATC; 48F:TCTTGGTGCACAGCGTGT; 49F:ACCCTCACGTGTACCGCT; 50F:TCCTTCCGCAAACACTTCCG; 51F:GCGGTGTACCAATCCAGGTG; 52F:CGCATACAATTTGGCCCTTGC; 53F:GGACCGTTCAGGCCCACTT; 54F:TTTCCAGTACGCACCGCAC; 55F:CGGTACTTGTGCGTCAGCTG; 56F:TCTCATCCACGGTGATGACGT; 57F:CTCGGCTTGCCTCGATGTG; 58F:CCTCTACTCTTGCAGACAGCC; 59F:ACAGGCGAGTGCCTCCAC; 60F:GGACTTGTGGCAGCACTC; 61F:TAGGTACAAGGCAGGGCTAAG; 62F:GTTGGAATCCGTGGAGAACC; 63F:AGAATAACCGGGGTCACAGC; 0R:CTGGGTATGACAGCGAGGTT; 1R:AAGAGAAGTAACACCTGCATGCT; 2R:CGGACCACAAGCCGCTATG; 3R:GATGTTGTGGCCACCGAGAC; 4R:TGTCGGGGACAGCGGAA; 5R:GTCCGTCTCGTTGCAGCAA; 6R:AGCCAAGACTCCGCACCA; 7R:CCAAGGTTGGCGCATTGC; 8R:GTCAAGCCCCTGAAGGTGATC; 9R:CGCAGGTCCAGAAAATGTCGT; 10R:TGTCCAAACCGTAACCAGAACTG; 11R:CTGTCATGGCGACCATCCT; 12R:GCATAACCAGCAGTTAATGAGACC; 13R:GCAAACCAGCTTACCCGTGA; 14R:CCGCCGTGGCCATAAACA; 15R:TTTCCGACACACAGCGAGC; 16R:TGCCACACACCACAACGTC; 17R:AGCCCATAGACATATGCAAAGCC; 18R:CGCAGCGGAAGTAAGCCAA; 19R:CCCCGCGTAAGGAGAAAGGA; 20R:GGCACCACTTGGCGTACA; 21R:CGAAGAGCCAGTCAATGAGGTC; 22R:CAAGTCGACAGCGCGGT; 23R:AGGGGGCTGTGAGAGTGTAC; 24R:TCGTCCCTTGGTGCCGT; 25R:CGTGCCATGAACCAGGGAAC; 26R:GGTTGCACCCTCCATGGAAC; 27R:CAACACACTGTGCCCGTCTT; 28R:TCCCTGGAGGAACCCTTGC; 29R:AGTGATGTTCTTGTGGGCAGC; 30R:GCTCTTCCCCCGCCAGTAT; 31R:GGGCCAACACTTACGCCT; 32R:AGTACCTAGTCACGTCGGCG; 33R:GCACACGTGGCGTTGTATG; 34R:CCCAAGGAGACGTCGTGGTA; 35R:GCGTCAGCTGGAATAGGCT; 36R:GCGTAGGGATTCCGTGCAC; 37R:CGCACACCAGCAACCACA; 38R:TCGGCCGTCGAAGAGGT; 39R:GCGAAGAAGCCGTCAGGAAG; 40R:CCGCGAAATCCAGCAGCA; 41R:CCCCAGGAAGAGCTGCAAAA; 42R:CGTGGCGAGCGACGTTT; 43R:GGGCATGTTTGGGAACTTCAC; 44R:TGCCCTGCGAGCCTCAA; 45R:CGACTCCTCAAGCTTCACCTTC; 46R:ACCACGCCCCGCATGAT; 47R:ACCTTTGGTGTGGAGAGTGC; 48R:CCCGTACCTAGGTTTTTCAGCTG; 49R:CCGTCATAGTAGGTTGCTGGC; 50R:CAAGTGTGCAGTCAGGGGG; 51R:CCGCCTCCTCGTACGAGTA; 52R:AAGTTGGTCGGGTTTGTGGG; 53R:CACAGGTACCTTCTTGGAGCG; 54R:ATGCCGGTAGCCCACGAT; 55R:TAGGGCGTCGCTCTCGT; 56R:ACAGGGTCTCCGTACTCAGC; 57R:GCTCCAAGCTATTACCTCGGAC; 58R:TTCACCTTGCGGCGCAC; 59R:GTCAACCACCTGTACCCGTG; 60R:CATGACTGGTTCAAGGACCCG; 61R:AGCCCGCAACACTCCGA; 62R:GGGGGTGTTCGTGAGAC; 63R:GCGCCGCCCTTAAGAACT. Additional primers, designed manually in Primer3_v.0.4.0 [39,40], were spiked into the primer pools to improve read depth in areas with reproducibly poor coverage: 4F.2:GCTCGGTCTTTCAGGTGTG; 6F.2:GACGTGGGCCTGGTCTTT; 7F.2:GAGGCGATGGACCTGTTTG; 54F.2:GTGGCGAAAGCTGTGTTGG; 61F.2:TCCTATCCCTAGTGGGGTGA; 4R.2:CCGCAGAGGAAAGCAAGG; 6R.2:CGTAGTACTCACCCAAGCCTA; 7R.2:AGGCACAGCTTGAACGAAAG; 42R.2:TGACAAGATCAAGCCTGTGC; 54R.2:GGGGCTCCCAGCATAGTG; 61R.2:CCCCACATCTGCGTTACTG. Illumina TruSeq adaptors were added to maPgV-specific primers and primers were ordered in “even” and “odd” pools based on sequential amplicon number so as to minimize interference between overlapping amplicons during PCR amplification (IDT, Coralville, IA). RT-PCR was performed on extracted maPgV RNA according to recommendations from PrimalScheme using the SuperScript IV One-Step RT-PCR System (Invitrogen, Waltham, MA). Following cleanup with Ampure XP beads (Beckman Coulter, Brea, CA), “even” and “odd” amplicon pools were combined for each sample and subjected to index PCR (UW Biotechnology Center). Paired-end 150-bp sequencing was performed using the NovaSeq6000 system (Illumina, San Diego, CA, USA).

#### Sequence analysis

Illumina sequencing data was imported into Geneious Prime (version 2022.2.2) (Biomatters, Auckland, New Zealand) as paired reads, then merged using the BBMerge tool Version 38.84. Merged reads were then mapped to the “consensus RPgV” sequence––this reference was used for all mappings and is comprised of the RPgV consensus sequence with the first 156 nucleotides originating from maPgV 5′ RACE and the last 151 nucleotides (nt 10637–10941 in the genome) originating from maPgV 3 ′ RACE. Read mapping was performed using Bowtie2 (v2.4.5) [41,42], with 20bp (for sequences generated via unbiased/SISPA) or 30bp (for sequences generated via PrimalScheme) trimmed from both ends prior to mapping to remove primers. Single nucleotide variants were then characterized using the built-in “find variations/SNPs” function in Geneious, using a minimum variant frequency of 0.05 and a minimum coverage of 100. Genbank Accession IDs for RPgV and maPgV are PP467554 and PP467555, respectively. Raw Illumina deep sequencing reads can be accessed in NCBI’s Short Read Archive (SRA) under accession numbers SAMN41664767–SAMN41664785 (BioProject ID PRJNA1119917).

#### RNA structural analysis

RNAfold [20] was used to predict the secondary structure and generate RNA structure scores (RSS) [21] of RPgV and maPgV genomes. Sliding windows of 200 nucleotides (step size = 1 nt) were folded (37°C) using RNAfold, and the RNA structure score (RSS; frequency of MFE (minimum free energy structure)/ensemble diversity) and z-score of each RSS was computed and plotted versus the nt window start size. RNA structure drawings of the minimum free energy structures were generated using RNAcanvas [43].

#### Histopathology and quantitative immunohistochemistry

Whole cross sections of spleen generated from formalin fixed paraffin embedded tissue were stained with hematoxylin and eosin, as well as by immunohistochemistry with an antibody directed against cleaved caspase 3 (Cell Signaling, #9579S). Positive IHC staining was quantified with QuPath Bioimage Analysis software v0.5.1 [44], data presented as percent positive cells in whole cross sections of quantified spleen. Raw datasets used to make each figure are uploaded Dryad [45].

#### Flow cytometry

Mice were euthanized via CO_2_ asphyxiation in compliance with approved University of Wisconsin–Madison Institutional Animal Care and Use Committee procedures (protocol M006443). Following euthanasia, spleens were harvested and immediately placed on ice, in tubes containing RPMI (Gibco), until processing. Spleens were then mashed using a syringe plunger and filtered through sterile 0.4-μm cell strainers (Fisher Scientific). The cell suspension was then pelleted and resuspended in ACK lysis buffer (Gibco) to lyse contaminating red blood cells. Cells were pelleted, rinsed with RPMI, then transferred to a 96-well V-bottom plate (Corning) and pelleted again prior to staining. Nonspecific Fc receptor binding to antibodies was blocked by resuspending cells in TruStain FcX (anti-mouse CD16/32) PLUS antibody (Biolegend) in FACS buffer (PBS + 2% FCS + 1mM EDTA) for 10 minutes at 4C. Without removing Fc block, antibodies (Table 3) were added to cell suspensions and incubated in the dark for 20 minutes at 4C at manufacturer’s recommended concentrations. Cells were rinsed in FACS buffer, pelleted, then fixed in 4% PFA for 30 minutes at room temperature. Samples were washed with FACS buffer, then filtered through a 100-μm nylon mesh prior to running on a Northern Lights full-spectrum flow cytometer (Cytek Biosciences, Bethesda, MD, USA). UltraComp beads (Invitrogen), unstained controls, and fluorescence minus one (FMO) controls were used for establishing gates and evaluating fluorophore interaction. Results were analyzed using FlowJo v10.8 software (BD Life Sciences).

**Table 3:** Flow cytometry reagents used in this study.

Fluorophore	Target	Clone	Manufacturer
none	Anti-mouse CD16/32	S17011E	Biolegend
PE	Anti-mouse CD4	RM4-5	Biolegend
PE-Cy7	Anti-mouse CD8a	53–6.7	Biolegend
Spark Violet 423	Anti-mouse CD3	17A2	Biolegend
KIRAVIA Blue 520	Anti-mouse CD20	SA275A11	Biolegend
APC	Anti-mouse CD45	I3/2.3	Biolegend

## Dryad DOI

https://doi.org/10.5061/dryad.h44j0zpv6 [45]

## Supporting information

S1 Fig Deep sequencing of maPgV. Illumina deep sequencing of RPgV at various points during mouse adaptation. The genome position of RPgV/maPgV is shown along the X-axis, with a schematic of predicted mature proteins shown in green across the top. The frequency of non-synonymous mutations (red) and synonymous variants >5% relative to the RPgV consensus sequence are shown along the left Y-axis, with a dashed black line denoting 50% frequency (i.e., consensus-level variants). Coverage is shown in gray on a log10 scale along the right Y-axis with a read-depth cutoff of 100 shown as a gray dashed line, below which variants were not called. Note that some samples were sequenced via unbiased deep sequencing and others were sequenced by multiplexed PCR amplicon sequencing, generating the “mountainous” versus “city-scape” appearing coverage plots, respectively.
(TIF)

S2 Fig AlphaFold3 model of glycosylated RPgV E1E2. High mannose glycans were modeled at each putative N-linked glycosylation site in E1E2 (E1: N22, N117 E2: N24, N40, N49, N101, N107 and N231). E1E2 is shown at two rotations with glycans labeled. E1:N117, which is lost in maPgV, is labeled in red.
(TIF)

S3 Fig ColabFold-AlphaFold2 models of RPgV and maPgV E1E2 heterodimers. Structures are color-coded by pLDDT (as in Fig 5). Curved lines indicate the approximate location of the viral membrane.
(TIF)
