# Supplementary figures and images for: Determinants of pegivirus persistence, cross-species infection, and adaptation in the laboratory mouse
Source: PLoS Pathog. 2024 Aug 28;20(8):e1012436. doi: 10.1371/journal.ppat.1012436 (PMC11355568; doi:10.1371/journal.ppat.1012436)

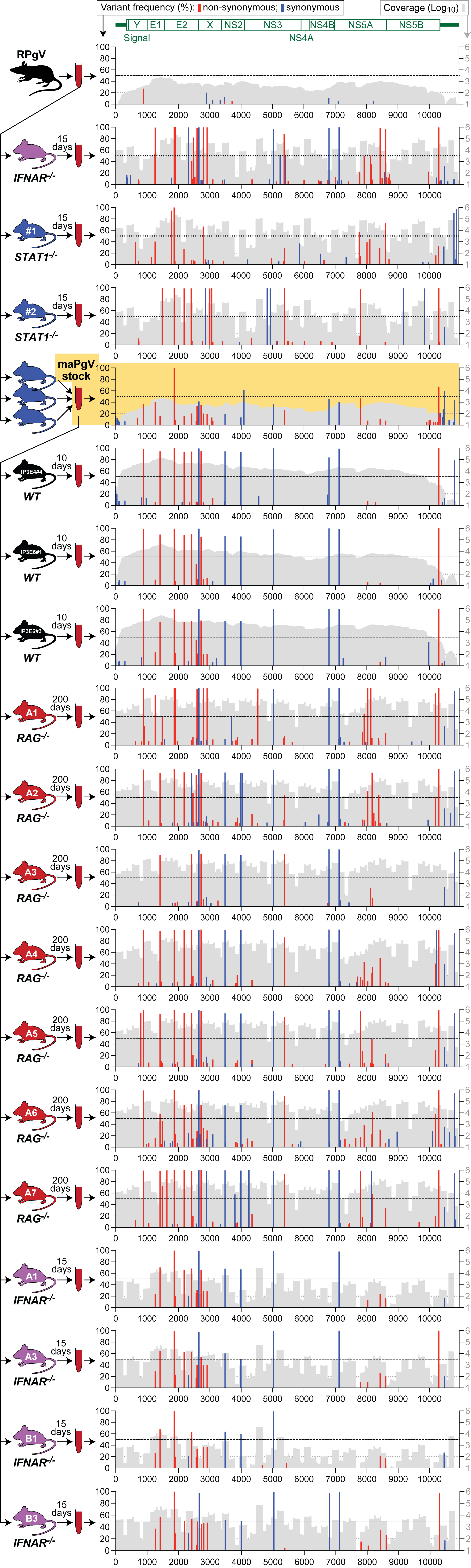

Supplement: S1 Fig — Illumina deep sequencing of RPgV at various points during mouse adaptation. The genome position of RPgV/maPgV is shown along the X-axis, with a schematic of predicted mature proteins shown in green across the top. The frequency of non-synonymous mutations (red) and synonymous variants >5% relative to the RPgV consensus sequence are shown along the left Y-axis, with a dashed black line denoting 50% frequency (i.e., consensus-level variants). Coverage is shown in gray on a log10 scale along the right Y-axis with a read-depth cutoff of 100 shown as a gray dashed line, below which variants were not called. Note that some samples were sequenced via unbiased deep sequencing and others were sequenced by multiplexed PCR amplicon sequencing, generating the “mountainous” versus “city-scape” appearing coverage plots, respectively. (TIF) [file ppat.1012436.s001.tif]

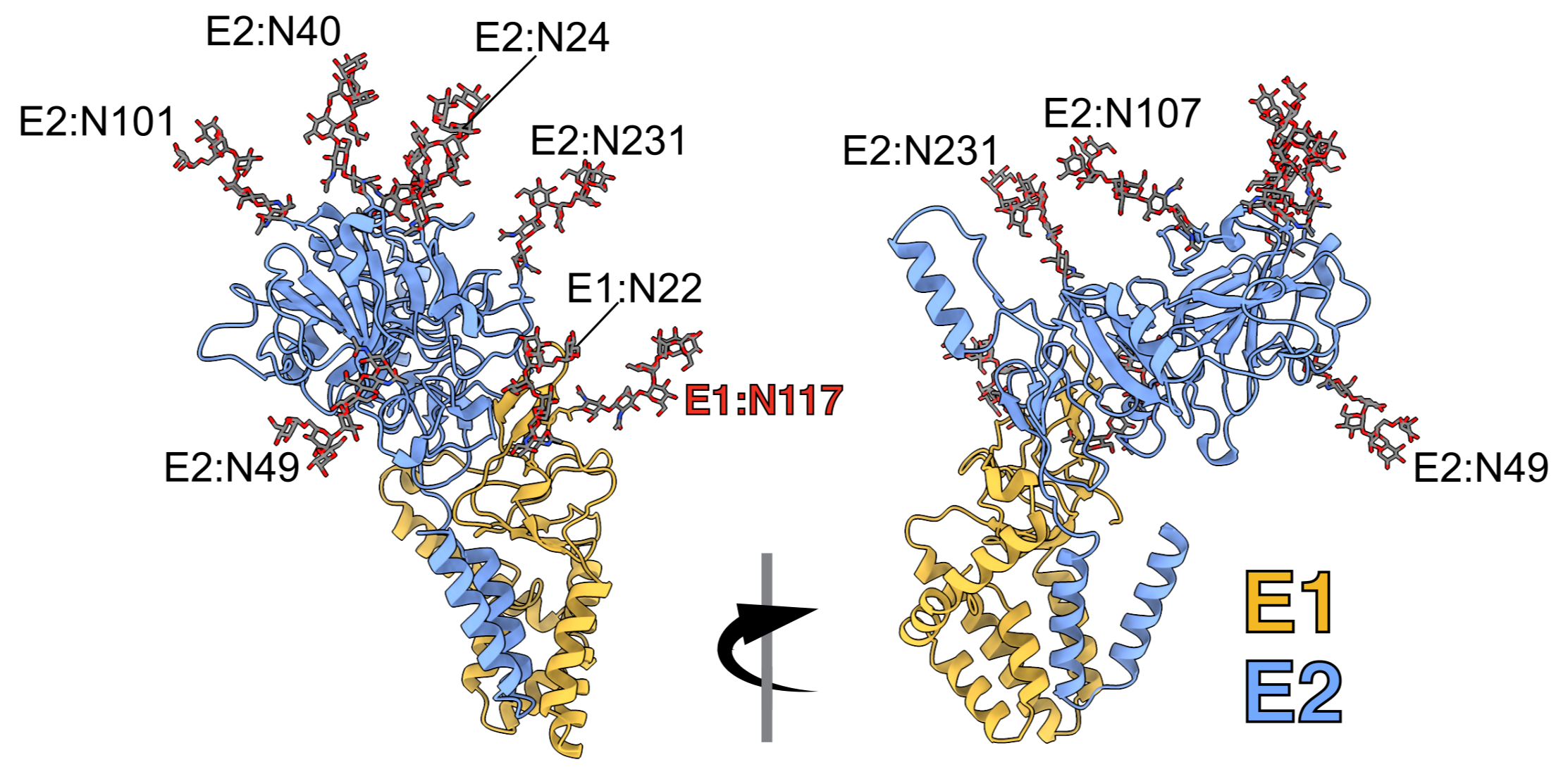

Supplement: S2 Fig — High mannose glycans were modeled at each putative N-linked glycosylation site in E1E2 (E1: N22, N117 E2: N24, N40, N49, N101, N107 and N231). E1E2 is shown at two rotations with glycans labeled. E1:N117, which is lost in maPgV, is labeled in red. (TIF) [file ppat.1012436.s002.tif]

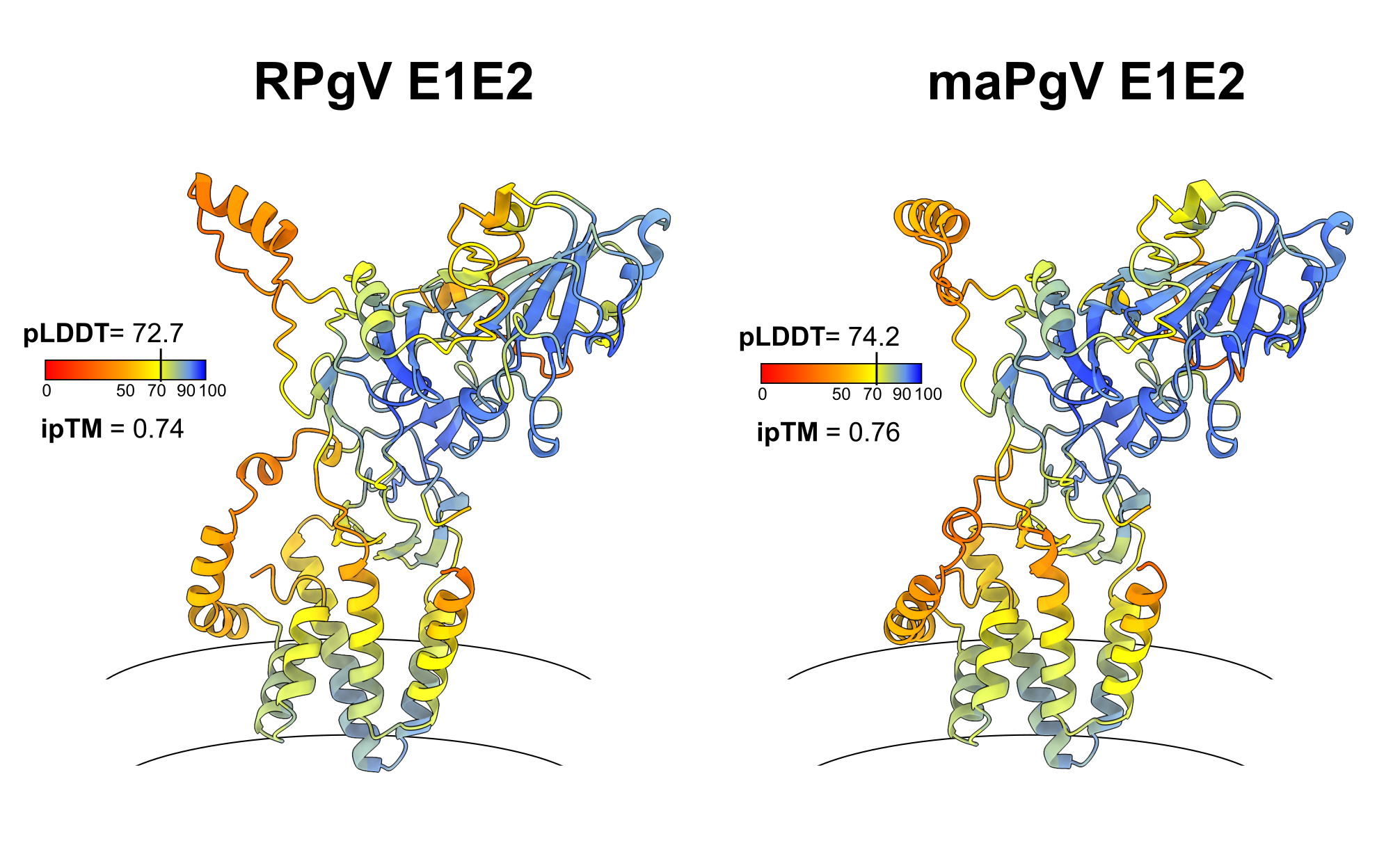

Supplement: S3 Fig — Structures are color-coded by pLDDT (as in Fig 5). Curved lines indicate the approximate location of the viral membrane. (TIF) [file ppat.1012436.s003.tif]
